# Treatment of myeloid malignancies relapsing after allogeneic hematopoietic stem cell transplantation with venetoclax and hypomethylating agents—a retrospective multicenter analysis on behalf of the German Cooperative Transplant Study Group

**DOI:** 10.1007/s00277-020-04321-x

**Published:** 2020-11-16

**Authors:** Esther Schuler, Eva-Maria Wagner-Drouet, Salem Ajib, Gesine Bug, Martina Crysandt, Sabine Dressler, Andreas Hausmann, Daniela Heidenreich, Klaus Hirschbühl, Matthias Hoepting, Edgar Jost, Jennifer Kaivers, Stefan Klein, Michael Koldehoff, Lambros Kordelas, Oliver Kriege, Lutz P. Müller, Christina Rautenberg, Judith Schaffrath, Christoph Schmid, Daniel Wolff, Rainer Haas, Martin Bornhäuser, Thomas Schroeder, Guido Kobbe, On behalf of the German Cooperative Transplant Study Group

**Affiliations:** 1 Department of Hematology, Oncology and Clinical Immunology, University Hospital Düsseldorf, Medical Faculty, Heinrich Heine University, Moorenstr. 5, 40225 Düsseldorf, Germany; 2 Department of Hematology, Oncology, Pneumology, Medical Clinic III, UCT Johannes Gutenberg-University Mainz, Mainz, Germany; 3 Department of Medicine II, Goethe University, Frankfurt am Main, Germany; 4 Department of Hematology, Oncology, Hemostasiology and Stem Cell Transplantation, Medical Clinic IV, University Hospital RWTH Aachen, Aachen, Germany; 5 Bone Marrow Transplantation Unit, Medical Clinic 5, Nürnberg, Germany; 6 Department of Hematology, Oncology, Immunology, Palliative Care, Munich Clinic Schwabing, Munich, Germany; 7 Medical Clinic III, University Medicine Mannheim, Mannheim, Germany; 8 Department of Hematology and Oncology, Medical Clinic II, University Hospital Augsburg, Augsburg, Germany; 9 Medical Clinic III, University Medicine Regensburg, Regensburg, Germany; 10 Clinic for Bone Marrow Transplantation, University Hospital Essen, Essen, Germany; 11 Department of Internal Medicine IV, University Hospital Halle, Martin-Luther-University Halle-Wittenberg, Halle, Germany; 12 Department of Internal Medicine I, University Hospital Carl Gustav Carus at the Technische Universität, Dresden, Germany

**Keywords:** Allogeneic hematopoietic stem cell transplantation, Relapse, Venetoclax, Hypomethylating agents, Azacitidine, Decitabine, DLI

## Abstract

Treatment of relapse after allogeneic hematopoietic stem cell transplantation (alloHSCT) remains a great challenge. Aiming to evaluate the combination of venetoclax and hypomethylating agents (HMAClax) for the treatment of relapse of myeloid malignancies after alloHSCT, we retrospectively collected data from 32 patients treated at 11 German centers. Venetoclax was applied with azacitidine (*n* = 13) or decitabine (*n* = 19); 11 patients received DLI in addition. HMAClax was the first salvage therapy in 8 patients. The median number of cycles per patient was 2 (1–19). All but 1 patient had grade 3/4 neutropenia. Hospital admission for grade 3/4 infections was necessary in 23 patients (72%); 5 of these were fatal. In 30 evaluable patients, overall response rate (ORR) was 47% (14/30, 3 CR MRD^neg^, 5 CR, 2 CRi, 1 MLFS, 3 PR). ORR was 86% in first salvage patients versus 35% in later salvage patients (*p* = 0.03). In 6 patients with molecular relapse (MR), ORR was 67% versus 42% in patients with hematological relapse (HR) (*n* = 24, *p* = n.s.). After a median follow-up of 8.4 months, 25 patients (78%) had died and 7 were alive. Estimated median overall survival was 3.7 months. Median survival of patients with HMAClax for first versus later salvage therapy was 5.7 and 3.4 months (*p* = n.s.) and for patients with MR (not reached) compared to HR (3.4 months, *p* = 0.024). This retrospective case series shows that venetoclax is utilized in various different combinations, schedules, and doses. Toxicity is substantial and patients who receive venetoclax/HMA combinations for MR or as first salvage therapy derive the greatest benefit.

## Introduction

Allogeneic hematopoietic stem cell transplantation (alloHSCT) is a curative treatment option in patients with myeloid malignancies. The most common cause of treatment failure after alloHSCT is relapse, and its treatment remains a great challenge [1]. In recent years, great effort has been made to evaluate targeted therapies, and by now gilteritinib and sorafenib as well as enasidenib are available [2]. Regardless of these achievements, for most patients relapsing after alloHSCT, either the targets are not present or targeted therapies have already been used for maintenance.

For patients with myelodysplastic syndromes (MDS) and acute myeloid leukemia (AML), 5-azacitidine (AZA) and less frequently decitabine (DAC) with or without donor lymphocyte infusions (DLI) have become a frequently used therapy for relapse after alloHSCT [3], with 2-year overall survival rates between 12 and 29% [4–8]. Further treatment options are intensive chemotherapy (IC) [9] or second alloHSCT [10, 11], which are toxic and show similar success rates [12]. Unfortunately, to our knowledge, randomized trials comparing these options have never been undertaken. Nevertheless, new, less toxic, and more efficient treatment options are urgently needed. The combination of venetoclax (VEN) and the hypomethylating agents (HMA) AZA or DAC has shown promising efficacy in elderly patients with AML, both as frontline therapy and for relapse [13–17]. Very recently first experience with patients proceeding to alloHSCT after this combination therapy was published with encouraging results [18]. Aiming to evaluate the combination of VEN and HMA (HMAClax) for the treatment of relapse of myeloid malignancies after alloHSCT, we retrospectively collected data from 32 patients treated from 11 German transplant centers and analyzed response, survival, treatment schedules, and adverse events.

## Methods

### Study design

The study included 32 patients who started treatment with the combination of an HMA and VEN until the end of May 2019 at 11 German transplant centers. There were no other inclusion criteria besides myeloid disease and treatment start. VEN was applied as off-label use therapy. Clinical data were gathered by the center for all patients that had been treated with HMA and VEN at the respective center and analyzed centrally in Duesseldorf. The study was approved by the local ethics committee of the Heinrich-Heine University Duesseldorf (study number 2019-541), and all patients gave informed consent on data collection and analysis. Adverse events were graded using National Cancer Institute Common Toxicity Criteria (NCI CTC) version 5.

Definitions of conditioning regimens regarding myeloablative (MAC) and reduced intensity (RIC) conditioning were in line with the European Society for Blood and Marrow Transplantation (EBMT) criteria [19]. Response criteria were used as defined by Cheson [20], disease risk was classified according to the ELN guideline [21], and GvHD according to NIH criteria [22–24]. Molecular relapse was defined as recurrence or increasing proportion of initial disease-specific molecular markers and/or loss of complete donor chimerism in peripheral blood or bone marrow. MRD was monitored by institutional standards according to established methods. Details of the 5 patients with molecular relapses are as follows: 2 were monitored with qPCR for NPM1, 1 for RUNX1/RUNX1-transkript, and 1 for WT1 and 1 with STR-PCR for loss of complete donor chimerism with a cut-off of 98%. In general, 2 independent markers of disease recurrence or confirmation with repeated analysis were required for the diagnosis of molecular relapse. The presence of more than 5% bone marrow blasts in line with decreasing donor chimerism was defined as hematologic relapse. Extramedullary relapse was defined as histologically or cytologically proven disease recurrence outside the bone marrow. DLI was defined as the infusion of donor blood cells without anteceding conditioning or immunosuppression.

### Statistics

All time-to-event variables were estimated by using the Kaplan-Meier method. For estimation of overall survival and survival after relapse, death from any cause was rated as an event. Surviving patients were censored at the last day of follow-up. For univariate analysis, we used the log-rank test to compare time-to-event curves from different groups and cross-tabulation with chi-square test for comparison of categorical variables. A *p* value of < 0.05 was considered significant. All data analyses were performed using SPSS 22.0 statistical software (SPSS Software GmbH, München, Germany).

## Results

### Patients’ characteristics

**Fig. 1:**
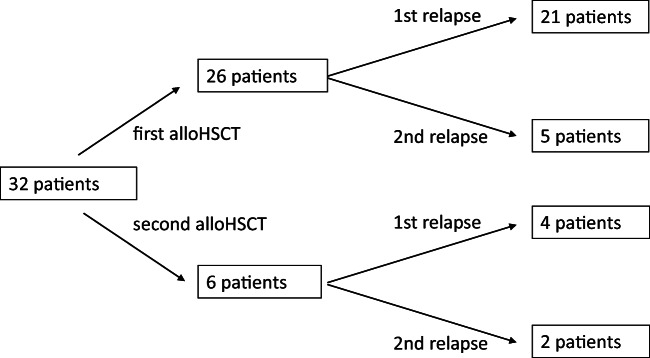
Timepoint of HMAClax-treated relapse. alloHSCT, allogeneic hematopoietic stem cell transplantation

Twenty-six patients were treated for relapse after their 1st and 6 after their 2nd alloHSCT (Fig. 1). Median time from alloHSCT to HMAClax–treated relapse was 5.7 months (1.1–67.8). Five patients had molecular relapses and 23 had hematologic relapses, 4 patients had extramedullary manifestations, 3 with concurrent hematological relapses, and 1 with molecular relapse. Diagnoses at the beginning of HMAClax therapy were AML in 30 patients and MDS in 2 patients. According to the European LeukemiaNet (ELN), classification of the majority of AML patients belonged to the high-risk group at the beginning of HMAClax therapy (*n* = 20), 6 patients belonged to the intermediate-risk group, and 4 to the low-risk group. Regarding cytogenetics at relapse, 11 patients had complex karyotypes, 4 involving 17p (TP53), and another 4 patients had molecular TP53 mutations. IDH2 mutations were present in 2 patients and NPM1 mutations in 5 patients including 1 patient with NPM1 mutation in combination with TP53, DNMT3a, and IDH1 mutations (patients´ characteristics are summarized in Table 1).

**Table 1:** Patients’ characteristics, *n* = 32

Male/female patients	50%/50%
Median age, years (range)	54 (30.8–71.5)
Preceding alloHSCT 1/2 (*n*)	26/6
Diagnosis at last alloHSCT	n
AML	26
MDS	4
CMML	1
aCML	1
CR/no CR at last alloHSCT (*n*)	9/23
Graft	n
MSD	4
MUD	21
Haploidentical family donor	7
MAC/RIC	15/17
Median time from alloHCST to REL, months (range)	5.7 (1.1–67.8)
Type of relapse	n
Molecular relapse (MR)	5
Hematological relapse (HR)	23
Extramedullary relapse (XR) (+HR/MR)	4 (3/1)
1st relapse after 1st alloHSCT	21
2nd relapse after 1st alloHSCT	5
1st relapse after 2nd alloHSCT	4
2nd relapse after 2nd alloHSCT	2
Diagnosis at HMAClax therapy	n
AML	30
MDS	2
ELN classification at HMAClax therapy for AML patients	n
High risk	20
Intermediate risk	6
Low risk	4
Cytogenetics at HMAClax therapy	
Complex karyotypes	11 (4 including 17p)
Molecular proven TP53 mutations	4
IDH 1 or 2 mutations	3*
NPM1 mutation	5*
Median blast count in bone marrow	20% (0–90)
Median white blood cell count/μl	4030 (500–220,000)
Median hemoglobin level (g/dl)	10 (6.9–15.4)
Median platelet count/μl	40,000 (1000–339,000)

*One patient had NPM1 mutation in combination with IDH1, TP53, and DNMT3a mutation

*alloHSCT* allogeneic hematopoietic stem cell transplantation, *AML* acute myeloid leukemia, *MDS* myelodysplastic syndrome, *CMML* chronic myelomonocytic leukemia, *aCML* atypical chronic myeloid leukemia, *CR* complete remission, *MSD* matched sibling donor, *MUD* matched unrelated donor, *MAC* myeloablative conditioning, *RIC* reduced intensity conditioning, *REL* relapse, *MR* molecular relapse, *HR* hematological relapse, *XR* extramedullary relapse, *ELN* European LeukemiaNet

Median time from relapse to HMAClax therapy was 1.8 months (0.3–42.9). HMAClax was the first-line therapy for relapse in 8 patients, second line in 22 patients, third line in 1 patient, and fifth line in 1 patient. In 21 patients, relapse had been refractory to HMA (± DLI, ± lenalidomide), 1 patient each had received lenalidomide, sorafenib, and DLI monotherapy prior to HMAClax therapy (Table 2). Eight patients were still on immunosuppressive therapy at the beginning of HMAClax therapy; immunosuppression was tapered during therapy. One patient received VEN prior to HMAClax-therapy during conditioning before alloHSCT.

**Table 2:** Pretreatment of HMAClax treated patients

Treatment line	n	Pretreatment	
first line	8		
second line	22	AZA DAC AZA + DLI AZA + Lena AZA + Lena +DLI Lena Sorafenib DLI	10 3 2 2 2 1 1 1
fourth line	1	AZA, DAC + DLI	
fifth line	1	DAC, ipilimumab, mitoxantrone, enasidenib	

*AZA 5* azacitidine, *DAC* decitabine, *DLI* donor lymphocyte infusion, *Lena* lenalidomide

### Treatment schedules and adverse events

Twelve patients received AZA and 19 DAC in combination with VEN. One patient received both HMA compounds as he was switched from AZAClax to DACClax because of rising minimal residual disease level (MRD) after 6 cycles and back to AZAClax after another 7 cycles. Eleven patients received DLI, 7 patients once, 2 patients twice, and 2 patient 3 times; median dose was 5 x 10^6^/kg/body weight (0.5–31). One patient with extramedullary relapse received radiation therapy and another one with extramedullary relapse had a complete surgical resection.

In 19 patients, VEN was planned as continuous therapy in a 28-day cycle and in 13 patients for 21 days with a drug-free period of 7 days before starting the next cycle. The starting daily dose varied between 20 and 400 mg and final doses ranged between 200 and 1600 mg; the highest continuous daily dose a patient received during a complete treatment cycle was 800 mg. A ramp-up period was planned in 15 patients in the first cycle.

Median number of cycles per patient was 2 (1–19). In total, 90 cycles were given, median duration of a cycle was 33 days (17–92), median VEN dose per cycle was 6400 mg (600–36,000), and median number of days with VEN was 21 (5–92). Five patients did not receive a complete cycle (VEN < 14 days). In total, 9 cycles were interrupted earlier than d14, 8 because of infection and 1 because of a grand mal seizure.

Three patients had non-fatal tumor lysis syndrome, although 2 of these had a ramp-up period. Characteristics of HMAClax therapy are summarized in Table 3.

**Table 3:** Characteristics of HMAClax therapy

Median time from REL to HMAClax, months (range)	1.8 (0.3–42.9)
Planned administration of VEN 21/28 d, n	13/19
Combination partner of VEN	n
AZA/DAC, n	12/19^#^
AZA/DAC + DLI, n	11
Median number of cycles per patient	2 (1–19)
Total number of cycles,	90
Median duration of cycle	33 days (17–92)
Number of days with VEN	21 days (5–92)
Non-fatal tumor lysis syndrome	3 patients

*REL* relapse, *HMA* hypomethylating agent, *HMAClax* HMA + venetoclax, *DLI* donor lymphocyte infusion, *VEN* venetoclax

All but 1 patient had grade 3/4 neutropenia and 26 patients (81%) had grade 3/4 thrombocytopenia at any time point of treatment. To better understand the contribution of HMAClax therapy to hematotoxicity, we analyzed all cycles that were started with neutrophils > 1000/μl (*n* = 47), of those 2 were ongoing. Of the remaining 45 cycles, recovery of neutrophils occurred in 39 cycles (87%) after a median time of 12 days (0–42). Since 18 of these cycles were given to one patient, we next analyzed all first cycles that were started with neutrophils > 1000/μl (*n* = 13). Recovery of neutrophils occurred in 9 cycles (69%) after a median time of 14 days (0–42). In only 6 of 35 (17%) cycles, which were started with neutropenia grade 3/4, recovery took place prior to the next cycle (median after 30 days (21–60)). Regarding thrombocytopenia, 51 cycles were started with a platelet count > 50.000/μl. Thrombocytopenia occurred in 8 (16%) of those cycles. In 4 of these 8, no recovery of platelets was seen. Duration of thrombocytopenia in the remaining 4 was 9, 15, 22, and 35 days. In the first cycle, 10 patients started with more than 50,000 platelets/μl. Among these 5 patients suffered from thrombocytopenia grade 3/4, 3 recovery took place after 15, 22, and 35 days. In the 34 cycles that were started with thrombocytopenia grade 3/4, recovery of platelets occurred in 3 (9%) after 22, 29, and 31 days.

Infection prophylaxis was given according to the respective internal standard of the center; 18 patients received azoles and 11 gyrase inhibitors. In patients receiving azoles or gyrase inhibitors, the VEN dose was adapted according to the VEN prescribing information. Hospital admission for grade 3/4 infections was necessary in 23 patients (72%). Five of these infections (22%) were fatal. There was no difference in frequency of severe infections between patients, who received an azole or a gyrase inhibitor and those that did not.

Eight patients were still on immunosuppression at the start of HMAClax. Of those 5 suffered from grade 3/4 infection (63%). So did 18 of 24 (75%) patients without immunosuppression (*p* n.s.). Three patients had aGvHD and 1 patient cGvHD prior to HMAClax-therapy, which persisted during treatment. One patient developed cGvHD during HMAClax therapy; however, this patient also received a DLI. One patient achieved hematologic remission of AML but simultaneously suffered from graft loss and autologous recovery during HMAClax-therapy.

### Response

Two patients died of infection before first response evaluation. In 30 evaluable patients, overall response rate (ORR) was 47% (14/30, 3 CR MRD^neg^, 5 CR, 2 CRi, 1 morphologic leukemia-free state (MLFS), 3 partial remission (PR)). Median time to best response was 1.5 months (0.7–4.2) or 1.5 cycles (1–4). Seven patients lost best response after a median of 2 months (0.4–3.6.), 2 underwent second transplant in remission, and 5 have ongoing responses (1.8, 3.8, 4.4, 7.4, and 12.9 months at last follow-up). Half of the responding patients received DLI (7/14).

Eight patients received HMAClax as first therapy for relapse; of those 7 were evaluable and 6 responded (ORR 86%), which was significantly better than patients who received HMAClax as later salvage therapy (8/23 (35%), *p* = 0.03). Three patients with first salvage therapy had a molecular relapses. Cross-tabulation of patients with molecular relapse in the first-line and the later salvage therapy group showed that the proportion of patients with molecular relapses was not significantly different between first and later salvage therapy groups. In total 6 patients had molecular relapses and 4 of these responded (67%); ORR in 24 patients with hematological relapses was 42% (*p* = n.s.). Six of 21 (29%) patients who received HMA prior to the HMAClax combination responded.

Regarding treatment schedule modalities, there was neither a difference regarding ORR between patients who received AZA or DAC, nor between DLI or no DLI, nor between 21 or 28 days with VEN administration.

Regarding disease risk and response, there was no difference between patients with more or less than 20% marrow blasts and patients with or without adverse genetics (TP53 mutation or complex karyotype). In detail, 7 of 16 (44%) patients with more than 20% blasts and 7 of 14 (50%) patients with less than 20 % blasts responded; 3 of 8 (38%) patients with TP53 mutation responded and 11 of 20 (55%) patients without (in 2 patients TP53 mutational status was unknown). Of 11 patients with a complex karyotype, 4 responded (36%) and of 19 without complex karyotype, 10 (53%) responded. Of the 4 patients with an isolated NPM1 mutation, 2 patients that were treated for molecular relapse achieved CR MRD^neg^, 1 died of infection before first response evaluation and 1 progressed. The two responding NPM1 molecular relapse patients have ongoing responses of 22.1 months and 7.4 months, the first patient lost and re-achieved response again with further treatment, another patient with t8;21; RUNX1-RUNXT1-translocation with molecular relapse who also received DLI has an ongoing response for 6 months. One of 2 patients with IDH2 mutations achieved CR and 1 progressed. Finally, time from transplant to relapse (>/< 6 months) had no significant impact on response.

### Survival

**Fig. 2:**
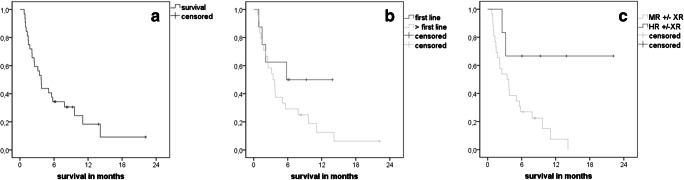
Kaplan-Meier Curves regarding overall survival: **a** all patients, **b** first salvage versus > first salvage therapy, **c** hematologic versus molecular relapses. **a** Median survival of all patients (*n* = 32) was 3.7 months (CI 2.8–4.6). **b** Median survival of patients receiving HMAClax as first (*n* = 8) vs > first salvage therapy (*n* = 24) was 5.7 vs 3.4 months (*p* = n.s.). **c** Median survival of patients with MR (*n* = 6) vs HR (*n* = 26) (± XR) was not reached vs 3.4 months, *p* = 0.024

On November 20, 2019, median follow-up was 8.4 months, 25 patients (78%) had died, and 7 were alive (Fig. 2). Four were continuing HMAClax therapy, 3 in best response and 1 lost best response. Estimated median overall survival time was 3.7 months (CI 2.8–4.6). Median survival of patients who received HMAClax as first-line therapy was 5.7 months and of patients who received HMAClax as later salvage therapy was 3.4 months (*p* = n.s.). Median survival of patients with molecular relapse was not reached vs 3.4 months for patients with hematological relapses (*p* = 0.024) (Fig. 2).

### Further therapy

Nine of 32 patients received further therapies. In 1 patient with persistent neutropenia on HMAClax therapy, VEN was continued as monotherapy; this patient is still in remission. In total, 4 patients received a subsequent transplant, 2 in remission after HMAClax-therapy, of these 1 is still in remission on d188 and the other died of relapse on d95. Both patients that were not in remission at subsequent transplant died of progression after transplant on d115 and d290, the latter after receiving intensive immunosuppressive therapy for GvHD. One patient each received enasidenib, anti-CD33 antibodies, cytarabine, and bortezomib and AMG 176 (MCL1-inhibitor), all of these died.

## Discussion

There is an unmet medical need for a safe and effective treatment strategy for relapse after alloHSCT for patients with myeloid malignancies. Regardless of emerging targeted treatment options for defined small groups of patients, treatment strategies for the majority of patients are DLI plus treatment with HMA or IC and in selected individuals a second alloHSCT. Although there is a remarkable success of these approaches in selected patients (e.g., late and molecular relapses), the majority of patients with early hematological relapse fails to achieve CR and long-term survival.

We here report a retrospective multi-center experience on the use of a combination of hypomethylating agents and the BCL2-inhibitor venetoclax for the treatment of relapse after alloHSCT. Earlier reports have included smaller groups of patients relapsing after alloHSCT so far [25–29].

In this case series, 32 patients have been treated with different combinations of HMA and VEN. The ORR was 47% for all patients. The disease was at different stages permitting the comparison of patients receiving HMAClax as first-line therapy for relapse with patients who had been pretreated with other salvage strategies which revealed significantly better response rate to HMAClax if applied as first-line approach for relapse (ORR 86% versus 35%, *p* = 0.03). The response rate in patients who had received HMA for relapse was below the ORR of all patients (29% vs 47%, *p* = n.s.). Whether the worse outcome in higher line patients is due to HMA pretreatment itself or just reflects greater chemoresistance is unclear. There was no survival benefit for first-line patients (5.7 versus 3.4 month; *p* = n.s.). Our analysis shows that early detection and treatment of molecular relapse improves the chance to achieve CR (ORR 67% versus 42%; *p* = n.s.) and survival (not reached versus 3.4 months; *p* = 0.024), when compared to overt hematological relapse, which is in line with earlier observation from others [30] and our center[31]. Consequently, we emphasize the urgent need for frequent MRD monitoring using highly sensitive molecular detection techniques. The higher response rate in the first-line group argues for applying HMAClax as early as possible once relapse has been confirmed.

Bryne et al reported an overall response rate of 9/16 (56%, CR, CRi, MLFS) for HMAClax [29], and Ram et al. a response in 4 of 6 patients (67% ORR) for HMAClax and DLI after alloHSCT, but information on treatment line was lacking [25]. In another small series with 4 heavily pretreated patients, 1 achieved CR and 1 a hematological response [28]. Responses were achieved very fast, in our group in 1.5 months, which is different from treatment with AZA or DAC plus DLI without VEN (2.8 and 5.2 months) [6, 8]. This data is in line with the time to response in the prospective trial for elderly people with HMA and VEN first line [32] and the relapsed/refractory patients [13]. Unfortunately, the duration of response was short in most of our cases. The combination of cytotoxic therapy combined with a cellular approach to induce an allo-immune effect has proved to be a successful approach. A study by the Chronic Malignancies Working Party of the EBMT analyzed the outcome of MDS and sAML after hematological relapses in patients receiving either cellular therapy (DLI or second alloHSCT) or chemotherapy and durable responses were seen almost exclusively with cellular therapy [33]. Therefore, a combination of HMAClax with DLI or subsequent transplant seems to be essential for long-term disease control. More information is needed on patients receiving HMAClax as first-line therapy in combination with DLI, to evaluate if this combination is able to produce durable responses for patients with hematological relapses as well. Extrapolating our experience with AZA or DAC in combination with DLI alone, we expect an equal or even better proportion of long-term responders. If no DLI is available, the feature of inducing fast but short responses in pretreated patients is well suited to be used prior a second alloHSCT in selected patients. However, we and others showed that CR of the underlying disease at the time point of second alloHSCT is not mandatory for long-term survival [31, 33].

Recently, molecular features have been described which were associated with a high probability of response [32]. One was the presence of a NPM1-mutation. In our cohort, both patients with NPM1-molecular relapse reached CR^MRD-^, which may be rather explained by relapse status being molecular than by the mutation itself. Regarding TP53, we did not observe a difference between mutated and not mutated patients.

Regarding GvHD, HMAClax seems to be safe; in our cohort, only 3 patients had ongoing aGvHD; in one of those immunosuppression was tapered simultaneously with HMAClax-therapy. One patient who also received DLI developed cGvHD and had to be treated with low-dose oral steroids. The immunologic effects of DLI seem not to be reinforced by HMAClax; however, others published single cases of GvHD after treatment with HMAClax and DLI [25, 27, 28].

Toxicity of HMAClax is substantial; all but 1 patient had severe neutropenia at 1 time point during treatment. As usual in this situation, toxicity and disease-related cytopenias especially in patients with hematological relapses were merging. In case of prolonged neutropenia, the use of granulocyte-colony stimulating factor may be justified. Despite antibiotic, antiviral, and antimycotic prophylaxis according to institutional standards, 3 quarters of patients suffered from grade 3/4 infections. Twenty-two percent of these were fatal, resulting in a mortality rate of 16% due to infections. The high rate of severe neutropenia and, as a result, life-threatening infection underlines the greater vulnerability of patients receiving HMAClax for relapse after alloHSCT in comparison to patients receiving HMAClax as first-line therapy (VIALE-A trial; neutropenia grade 3/4 42% and grade 3/4 infection 64%) [17]. Other investigators reported that 19% of patients in the relapsed/refractory cohort suffered from invasive fungal infections emphasizing the need for intensive prophylaxis and surveillance [34].

Tumor lysis syndrome was rare (3/32; 9%). A recent publication suggested that white blood cell counts should be lowered to below < 25.000/μl in general and in fast responding patients with IDH or NPM1 mutations even below 10.000/μl [2] before starting with VEN therapy. The authors further recommend a 3-day ramp-up of VEN.

Regarding the best partner and the best dose, we were not able to show an advantage for either of the HMA or an application model (with or without drug free period). Due to the non-standardized conditions of this retrospective analysis, no clear recommendations for dose and duration of VEN therapy can be made. These have to be established in a prospective trial. The phase III study for first-line treatment of AML patients, who are not able to tolerate intensive treatment, uses 400 mg as a daily dose [32]. Exposure-response data support 400 mg VEN daily dose as reasonable in combination with HMA [35]. From our retrospective data, we were not able to show a difference between a 21-day and a 28-day VEN schedule. All but 1 center applied the therapy in an outpatient setting. Since tumor lysis syndrome was rare; this procedure seems reasonable maybe with the exception of patients with NPM1 or IDH mutations [2].

In summary, this retrospective series shows that today venetoclax is utilized in various different combinations, schedules, and doses for the treatment of relapse of myeloid malignancies after alloHSCT. Toxicity is substantial and patients who receive venetoclax/HMA combinations for molecular relapse or as first salvage therapy derive the greatest benefit.

Future analyses will probably also contain more patients who received venetoclax containing regimens before alloHSCT, and a second challenge may be less effective in these cases.

Controlled prospective studies are needed to define the best partner, dose, and schedule of venetoclax in this particular setting including ancillary studies which focus on the interaction of BCL-2 inhibition with allogeneic immune responses.
